# The Clinical and Etiological Characteristics of Influenza-Like Illness (ILI) in Outpatients in Shanghai, China, 2011 to 2013

**DOI:** 10.1371/journal.pone.0119513

**Published:** 2015-03-30

**Authors:** Yifei Fu, Lifeng Pan, Qiao Sun, Weiping Zhu, Linying Zhu, Chuchu Ye, Caoyi Xue, Yuanping Wang, Qing Liu, Ping Ma, Huifang Qiu

**Affiliations:** 1 Research Base of Key Laboratory of Surveillance and Early Warning on Infectious Disease in China CDC, Shanghai Pudong New Area Center for Disease Control and Prevention, Shanghai, China; 2 Department of Microbiology Laboratory, Shanghai Pudong New Area Center for Disease Control and Prevention, Shanghai, China; University of Minnesota, UNITED STATES

## Abstract

**Introduction:**

Clinical and etiological characteristics of influenza-like illness (ILI) in outpatients is poorly understood in the southern temperate region of China. We conducted laboratory-based surveillance of viral etiology for ILI outpatients in Shanghai from January 2011 to December 2013.

**Materials and Methods:**

Clinical and epidemiological data from ILI outpatients, both children and adults, were collected. A total of 1970 nasopharyngeal swabs were collected and tested for 12 respiratory viruses using multiplex RT-PCR, and the data were analyzed anonymously.

**Results:**

All 12 respiratory viruses were detected in the specimens. At least one virus was detected in 32.4% of 1970 specimens analyzed, with 1.1% showing co-infections. The most frequently detected agents were influenza A (11.7%), influenza B (9.6%), and rhinoviruses (3.1%).Other viruses were present at a frequency less than 3.0%. We observed a winter peak in the detection rate in ILI patients during 3 years of surveillance and a summer peak in 2012. HCoV, HADV, and HMPV were detected more frequently in children than in adults. Patients infected with influenza virus experienced higher temperatures, more coughs, running noses, headaches and fatigue than patients infected with other viruses and virus-free patients (p<0.001).

**Conclusions:**

The spectrum, seasonality, age distribution and clinical associations of respiratory virus infections in children and adults with influenza-like illness were analyzed in this study for the first time. To a certain extent, the findings can provide baseline data for evaluating the burden of respiratory virus infection in children and adults in Shanghai. It will also provide clinicians with helpful information about the etiological patterns of outpatients presenting with complaints of acute respiratory syndrome, but further studies should be conducted, and longer-term laboratory-based surveillance would give a better picture of the etiology of ILI.

## Introduction

Influenza-like illness (ILI), a subset of acute respiratory infections (ARI), represents approximately 62% of acute respiratory infections[1]. Viral pathogens are the most significant contributors [2].ARI is caused by many viruses, including the following: influenza viruses (IAV, IBV and ICV), respiratory syncytial virus(RSV),rhinovirus(HRV), human parainfluenza viruses(HPIV)1–4, human metapneumovirus(HMPV), human coronaviruses (HCoV) NL63,229E,OC43, and HKU1, human enterovirus (HEV) and adenovirus(HADV)[3].The similar clinical presentations of patients infected by various respiratory viruses make etiological diagnoses difficult when physicians simply make decisions based only on physical symptoms. The etiology of influenza-like illness (ILI) has been well characterized in some parts of the world, especially in temperate regions of the Northern Hemisphere [4–7].However, much less is known about the etiology of ILI in China, especially in Southeast China, which is located in a temperate region.

Pudong New Area is the largest and most developed district in Shanghai, with an extremely dense population of 5.5 million in 2013, representing one-fifth of the population of Shanghai. Shanghai Dongfang Hospital and Shanghai Zhoupu Hospital have been national influenza surveillance sentinel units since 2007. As in other regions of China, ILI data from hospitals can provide valuable information that could be used for monitoring the onset of an epidemic, especially the circulation of influenza virus. However, the epidemiology in Pudong of viral etiology for ILI is poorly understood. With support from the Chinese Key SciTech Program for Infection Control(under The Twelfth Five-year Framework), a laboratory-based influenza-like illness surveillance system was established in 2011 in Pudong New Area. We adapted multiplex RT-PCR assays for the detection of influenza virus and a number of other respiratory viruses that have recently been introduced, and these assays are also more sensitive than culture [8–10]. The objective of this study was to examine the clinical and etiological characteristics of influenza-like illness (ILI) in outpatients in this area from January 2011 through December 2013.

## Materials and Methods

### Ethics statement

This study did not involve any health-related patient interventions. The surveillance and sampling protocols were approved by Shanghai Pudong New Area Center for Disease Control and Prevention Review Board. A verbal informed consent was obtained from each subject; however, the patient identities have not been disclosed at any stage. The institutional board was informed of the specific needs with reference to the study setting and approved this mode of consent. A check box was included in the data form to document the consent-taking procedure.

### Case definition and study population

An ILI case was defined as a sudden onset of fever(≥38°C), cough and/or sore throat, with no laboratory-confirmed evidence of etiology[11, 12].A co-infection was diagnosed when a patient tested positive for two viruses on RT-PCR. The study population, including children (<15 years) and adults (≥15 years),consisted of the ILI cases who sought medical attention in the outpatient fever clinics of the above mentioned two hospitals from January 2011 to December 2013.

### Specimens and data collection

Five to ten ILI cases per hospital who had not received antiviral drugs were invited to participate in this study each week. Nasopharyngeal swabs were collected by well-trained nurses. The swabs were sent in 3 ml of viral transport medium to Pudong New Area Center for Disease Control and Prevention within 48 hrs after collection and were stored at -70°C. Data were collected simultaneously using a standardized case report form, which included both demography and clinical information (such as symptoms, treatment, and temperature).

### Nucleic acid extraction

Nucleic acid was extracted by the NucliSens easy MAG platform with the NucliSens magnetic extraction reagents (bio Me´rieux, The Netherlands) according to the manufacturer’s instructions using a sample volume of 250μl. The elution volume of nucleic acid was 50μl.

### cDNA synthesis

Reverse transcription was performed with an ABI 9700 GeneAmp PCR system (Applied Biosystems, Singapore) using the first-strand cDNA synthesis reagents (Ferments, Germany), according to the Seeplex RV12 ACE Detection Kit’s user manual (Seegene Inc., Seoul, Korea). The final reaction volume was 20μl with 8μl total RNA, 1μl random hexamer (0.2μg/μl), 3μl nuclease-free water, 4μl 5×RT buffer, 2μl 10 mM dNTP, 1μl Rnase inhibitor (20 u/μl), and 1μl reverse transcriptase (200 u/μl). The reaction was chilled on ice for 2 min, run at 37°C for 90 min, 94°C for 2 min and chilled on ice for 2 min. All cDNA samples were stored at -20°C until use.

### RT-PCR and Real-time PCR

PCR amplification was performed with an ABI 9700 GeneAmp PCR system using a Seeplex RV12 ACE Detection Kit. It is designed for simultaneous detection of 12 respiratory viruses, including IAV, IBV, RSVA, RSVB, HADV, HMPV, HPIV1-3, HRV, HCoV-229E/NL63, and HCoV-OC43/HKU1. Sample cDNAs were tested in a 2-tube reaction following the protocol supplied by the manufacturer. HMPV, HADV, HCoV-229E/NL63, HPIV-2, HPIV-3 and HPIV-1 were detected by set A. IAV IBV, HCoV-OC43HKU1, HRV-A/B, RSV-A, and RSV-B were detected by set B. Positive controls included a mixture of all 12 virus clones. The negative control contained only ddH^2^O. Each set consisted of 4μl 5×RV12 ACE PM (A or B), 3μl 8-Mop Solution, 10μl 2×Multiplex Master Mix and 3μl of the sample’s cDNA. The PCR reaction program was 94°C for 15 min, 40 cycles of 94°C for 0.5 min, 60°C for 1.5 min and 72°C for 1.5 min, with extension at 72°C for 10 min.

Real-time PCR for human influenza C (IFVC) and human bocavirus(HBoV) was performed using an Influenza Virus C Real Time RT-PCR kit (Shanghai ZJ Bio-Tech Co., Ltd., People’s Republic of China)and a Diagnostic Kit for Human Bocavirus DNA (GuangzhouDAAN Gene Co., Ltd., People’s Republic of China). In accordance with the manufacturer’s instructions, PCR for ICV was performed in a volume of 25μl using ABI 7500 fast (ABI, USA). Each reaction consisted of 19μl IFVC Super Mix, 1μl RT-PCR Enzyme Mix and 5μl Extraction RNA template for a final volume of 25μl. The PCR reaction was performed in the instrument at 45°C for 10 minutes (min), and 95°C for 15 min, followed by amplification consisting of 40 cycles of 15 seconds (s) at 95°C and 1 min at 60°C. Positive and no-template controls were included in each run.PCR for HBoV was performed in a volume of 20μl using ABI 7500 fast. Each reaction consisted of 14μl HBoV PCR Reaction Mix, 0.7μl Enzyme Mix, 3.3μl Primer and Probe Mix and2μl Extraction DNA template for a final volume of 20μl. The PCR reaction was performed in the instrument at50°C for 8 min, 93°C for 2 min, 10 cycles of 45 s at 93°C and 1 min at 55°C, followed by amplification consisting of 30 cycles of 30 s at 93°C and 45 se at 55°C. Positive and no-template controls were included in each run.

### Electrophoresis

PCR products were sized using QIAxcel DNA Screening gel cartridge (QIAGEN) on the QIAxcel system (QIAGEN, Switzerland), which enabled high-resolution capillary electrophoresis. A 50–800 bp QX DNA Size Marker (Qiagen) was included on QIAxcel runs, and the size of the products was determined using the QIAxcel ScreenGel software (Qiagen). The QIAxcel system produces a digital gel image and an electropherogram for fragment analysis. The expected PCR product sizes of set A were 749bp (HMPV), 534 bp (HADV), 375 bp (HCOV-229E/NL63), 264 bp (HPIV-2), 188 bp HPIV-3), and 139 bp (HPIV-1), while the product sizes of set B were 754 bp (IFV-B), 578 bp (HCOV-OC43/HKU1), 394 bp (HRV-A/B), 273 bp (HRSV-A), 206 bp (IFV-A) and 143 bp (HRSV-B).

### Statistical Analysis

Data were collected by the staff of the sentinel hospitals and laboratories on a standardized case report form and entered into an online data management system established by China CDC. The data were analyzed with SPSS software. The mean and SD or median and IQR were calculated for continuous variables, and percentages were calculated for categorical variables. A Pearson chi-square test and one-way ANOVA were used for categorical data or continuous data, respectively, wherever appropriate. A multivariate unconditional logistic regression analysis was used to determine factors associated with various clinical signs of ILI in outpatients. All statistical tests were 2-sided. P values<0.05 were considered statistically significant.

## Results and Discussion

### Clinical characteristics of the ILI case-outpatients

A total of 1970 ILL outpatients who met the ILI-case definition criteria were enrolled in this study. Of these outpatients, 51.8% were male. The mean age was 19.7±16.9 years. A temperature reading of >39°C was documented in 262(13.3%) of the subjects. A total of 67.9% of the subjects reported a cough, 51% pharyngalgia, 40.5% running nose, 32.4% fatigue, 28.2% headache, and 7.6% myalgia. Patients infected with influenza virus experienced higher temperatures and more coughs, running noses, headaches and fatigue than patients infected with other viruses or patients who were virus-free(p <0.001) (Table 1).

**Table 1:** Clinical characteristics of ILI-outpatients in Shanghai from January 2011 to December 2013.

Characteristics	Influenza virus-positive	Other virus-positive *	virus-negative	P value	Total#
	n = 393	n = 245	n = 1332		n = 1970
Demographics					
Male	185(47.1%)	129(52.7%)	706(53%)	0.113	1020(51.8%)
age(y)(means±SD)	24.2±18.0	17.4±17.8	18.8±16.1	**<0.001**	19.7±16.9
Clinical					
Presentation					
Temperature(°C)≥39°C	79(20.2% of 391)	23(9.4% of 245)	79(5.9% of 1329)	**<0.001**	262(13.3% of 1965)
Cough	320(81.4%)	174(71.0%)	843(63.3%)	**<0.001**	1337(67.9%)
Sore throat	214(54.5%)	133(54.3%)	658(49.4%)	0.117	1005(51.0%)
Running rose	200(50.9%)	109(44.5%)	489(35.7%)	**<0.001**	798(40.5%)
Headache	139(35.4%)	60(24.5%)	357(26.8%)	**0.002**	556(28.2%)
Fatigue	161(41.0%)	67(27.3%)	410(30.8%)	**<0.001**	638(32.4%)
Interval (days)	1(0–1)	1(0–1)	1(0–1)	0.605	1(0–1)
Routine examination					
White cell count(×10^9^/l)	8.84±11.97(n = 358)	8.49±5.20(n = 219)	8.27±7.15(n = 1213)	0.496	8.41±8.16(n = 1790)
Percentage of neutrophis(%)	60.81±18.91(n = 351)	59.4±18.47(n = 216)	59.4±18.12(n = 1192)	0.439	59.7±18.32(n = 1759)
Percentage of lymphocytes (%)	25.5±22.7(n = 339)	29.1±20.0(n = 211)	28.5±19.6(n = 1162)	0.038	27.9±20.3(n = 1712)

Values are mean with SD or n (%) of outpatients unless otherwise stated. Normally distributed data are reported as means with SD and non-normally distributed data as medians with inter quartile range.

The characteristics contain demography characteristic, presenting symptoms, and clinical findings.

*Positive cases for at least one respiratory virus except Influenza viruses by RT-PCR.

# Include all ILI cases regardless of virology RT-PCR test results.

Boldface font indicates statistical.

### Viral etiology of ILI cases

All the subjects’ samples were analyzed (Table 2). All of the 12 respiratory viruses were detected. At least one agent was detected in 638 specimens (32.4%) by multiplex RT-PCR. The viruses most commonly detected were influenza A virus (IAV) and influenza B virus (IBV) at 230 (11.7%) and 162 (8.2%), respectively. All of the others were present at a frequency less than 4%: HRV in 61(3.1%) patients, HADV in 53(2.7%) patients, HCoV in 51(2.6%) patients, HPIV in 48(2.4%) patients, RSV in 47(2.3%) patients, HMPV in 5(0.3%) patients, and HBoV in 2(0.1%) patients (Table 2)A single infection was identified in 616 (31.3%) patients, and co-infection was observed in 22 (1.1%). There were 14 cases with co-infection in children less than 15 years of age, not significantly different (*p* >0.05) from the corresponding result for adults(≥15)(Table 3).Of the 22 co-infection specimens, the most frequent combination was IAV and HRV (n = 6), followed by IAV and HPIV, IBV and HCoV, HPIV and HCoV, RSV and HPIV, RSV and HCoV. Each of these combinations was observed in two cases. The combinations IBV and HRV, HPIV and HRV, RSV and HRV, RSV and HADV, IBV and HPIV, and HRV and HCoV were each observed in one case.

**Table 2:** Distributions of agents detected in respiratory samples in Shanghai Pudong, January 2011 through December 2013.

Month	Total samples	No.(%) of isolates													
		IAV	IBV	ICV	HPIV-1	HPIV-2	HPIV-3	RSV-A	RSV-B	HCOV-OC43/HKU1	HCOV-229E/NL63	HADV	HRV-A/B	HMPV	HBoV
Jan-11	42	21(50.0)	3(7.1)	0	0	0	0	0	0	0	0	0	0	0	0
Feb-11	46	2(4.3)	6(13.0)	0	1(2.2)	0	0	1(2.2)	0	1(2.2)	0	2(4.3)	0	0	0
Mar-11	44	0	17(38.6)	0	0	0	1(2.3)	0	0	1(2.3)	0	2(4.6)	0	0	0
Apr-11	30	0	5(16.7)	0	2(6.6)	0	0	0	0	0	0	3(10)	0	0	0
May-11	39	0	3(7.6)	1(2.6)	2(5.2)	0	3(7.6)	0	0	0	0	4(10.3)	0	0	0
Jun-11	32	0	0	0	0	0	0	0	0	0	0	2(6.3)	0	0	0
Jul-11	44	0	2(4.5)	0	0	0	1(2.3)	0	0	1(2.3)	0	3(6.8)	0	0	0
Aug-11	72	0	2(2.8)	0	2(2.8)	1(1.4)	1(1.4)	0	0	3(4.2)	1(1.4)	3(4.2)	0	0	0
Sep-11	88	0	3(3.4)	0	4(4.5)	4(4.5)	0	2(2.3)	4(4.5)	5(5.7)	11(12.6)	0	0	0	0
Oct-11	89	0	0	0	1(1.1)	2(2.2)	0	12(13.6)	0	0	4(4.5)	2(2.2)	0	0	0
Nov-11	28	0	3(10.7)	0	1(3.6)	2(7.1)	0	1(3.6)	0	0	0	2(7.1)	0	0	0
Dec-11	6	0	5(83.3)	0	0	0	0	0	0	0	0	0	0	0	0
Jan-12	145	0	53(36.6)	0	0	0	0	2(1.4)	0	0	1(0.7)	4(2.8)	0	0	0
Feb-12	120	9(7.5)	43(35.8)	0	0	0	0	2(1.7)	0	0	0	1(1.7)	1(1.7)	0	0
Mar-12	109	18(16.5)	14(12.8)	0	1(0.9)	0	0	2(1.8)	2(1.8)	1(0.9)	1(0.9)	2(1.8)	3(2.8)	0	0
Apr-12	79	12(15.2)	0	0	3(3.8)	0	0	0	1(1.3)	0	3(3.8)	3(3.8)	2(2.5)	1(1.3)	0
May-12	89	4(4.5)	1(1.1)	0	0	0	1(1.1)	1(1.1)	0	0	5(5.6)	0	4(4.5)	0	0
Jun-12	81	3(3.7)	2(2.5)	0	0	0	0	0	0	2(2.5)	3(3.7)	0	5(6.2)	1(1.2)	0
Jul-12	118	63(53.4)	0	0	0	0	0	0	0	1(0.8)	3(2.5)	0	5(4.2)	2(1.7)	0
Aug-12	57	16(28.2)	0	0	0	0	0	1(1.7)	0	0	0	0	1(1.7)	0	1(1.7)
Sep-12	49	5(10.2)	0	0	0	0	0	2(4.1)	0	0	0	0	6(12.2)	0	0
Oct-12	46	0	0	0	0	0	0	0	0	0	0	0	6(13.0)	0	0
Nov-12	48	0	0	0	0	0	0	1(2.1)	3(6.3)	0	3(6.3)	0	8(16.6)	0	0
Dec-12	74	17(22.8)	0	0	0	0	0	0	5(6.8)	0	0	1(1.4)	3(4.1)	0	0
Jan-13	72	11(15.3)	0	0	0	0	0	0	4(5.6)	0	0	5(6.9)	1(1.4)	0	0
Feb-13	19	5(26.3)	0	0	0	0	0	0	0	0	0	0	1(5.3)	0	0
Mar-13	20	6(30.0)	0	0	0	1(5.0)	0	0	0	0	0	1(5.0)	2(10.0)	0	0
Apr-13	19	3(15.8)	0	0	0	0	0	0	0	0	0	3(15.8)	2(10.5)	0	0
May-13	26	2(7.7)	0	0	1(3.9)	1(3.9)	2(7.6)	1(3.9)	0	0	0	1(3.8)	4(15.4)	0	0
Jun-13	16	0	0	0	0	0	0	0	0	0	0	1(6.3)	2(12.5)	0	0
Jul-13	51	5(9.8)	0	0	1(2.0)	3(5.9)	1(2.0)	0	0	0	0	4(7.8)	0	0	0
Aug-13	52	2(3.9)	0	0	1(1.9)	1(1.9)	0	0	0	0	0	2(3.8)	1(1.9)	0	0
Sep-13	38	2(5.3)	0	0	2(5.3)	1(2.6)	0	0	0	1(2.6)	0	1(2.6)	4(10.5)	1(2.6)	0
Oct-13	18	0	0	0	0	0	0	0	0	0	0	0	0	0	0
Nov-13	26	6(23.1)	0	0	0	0	0	0	0	0	0	1(3.8)	0	0	0
Dec-13	38	18(47.4)	0	0	0	0	0	0	0	0	0	0	0	0	12.6)
Total	1970	230(11.7)	162(8.2)	1(0.05)	22(1.1)	16(0.8)	10(0.5)	28(1.4)	19(1.0)	16(0.8)	35(1.8)	53(2.7)	61(3.1)	5(0.3)	2(0.1)

IAV: Influenza A virus; IBV: Influenza B virus; ICV: Influenza C virus; HRV: Human Rhinovirus; HADV:Human Adenovirus; HCoV-229E: Human Coronavirus 229E;HCoV-OC43: Human Coronavirus OC43; HPIV-1: Human Parainfluenza Virus-1; HPIV-2: Human Parainfluenza Virus-2; HPIV-3: Human Parainfluenza Virus-3; RSV-A: Respiratory Syncytial Virus-A; RSV-B: Respiratory Syncytial Virus-B;HMPV: Human Metapneumovirus;

**Table 3:** Distribution of Viral Etiology of the outpatients with influenza-like infection by age and gender, Shanghai, China, 2011–2013.

	0–4y		5–14y		15–24y		25–59y		≥60		P value	Male		Female		P value	Total	
	n = 105	%	n = 200	%	n = 91	%	n = 213	%	n = 29	%		n = 314	%	n = 324	%		n = 638	%
IAV※	25#	23.88§	65	32.5	40	44.0	89	41.8	11	37.9	**0.001**	121	38.5	109	33.7	0.833	230	36.0
IBV	9	8.6	55	27.5	23	25.3	70	32.9	5	17.2	**<0.001**	64	20.4	98	30.2	**0.001**	162	25.4
ICV	1	1.0	0	0	0	0.0	0	0.0	0	0.0		0(0)	0.0	1	0.3		1	0.2
HRV	14	13.3	21	10.5	9	14.3	15	7.0	2	6.9	0.77	33	10.5	28	8.6	0.712	61	9.6
HADV	16	15.2	19	9.5	6	9.5	12	5.6	0	0.0	0.065	30	9.6	23	7.1	0.476	53	8.3
HCoV-229E	14	13.3	10	5	2	3.2	6	2.8	3	10.3	**0.002**	19	6.1	16	4.9	0.764	35	5.5
HCoV-OC43	8	7.6	4	0.2	1	1.6	2	0.9	1	3.4	**0.01**	8	2.5	8	2.5	0.886	16	2.5
HPIV-1	6	5.7	5	2.5	4	6.3	5	2.3	2	6.9	0.342	12	3.8	10	3.1	0.794	22	3.4
HPIV-2	3	2.9	5	2.5	4	6.3	2	0.9	2	6.9	0.187	10	3.2	6	1.9	0.389	16	2.5
HPIV-3	1	1.0	2	1	1	1.6	4	1.9	2	6.9	0.067	5	1.6	5	1.5	0.91	10	1.6
RSV-A	6	5.7	12	6	1	1.6	7	3.3	2	6.9	0.335	12	3.8	16	4.9	0.341	28	4.4
RSV- B	6	5.7	5	2.5	2	2.2	4	1.9	2	6.9	0.205	10	3.2	9	2.8	0.94	19	3.0
HMPV	2	1.9	3	1.5	0	0.0	0	0.0	0	0.0		2	0.6	3	0.9	0.598	5	0.8
HBoV	1	1.0	1	0.5	0	0.0	0	0.0	0	0.0		1	0.3	1	0.3	0.96	2	0.3
Single infection	98	93.3	193	96.5	89	97.8	210	98.6	27	93.1		302	96.2	314	96.9	0.099	616	96.6
Co-infection	7	6.7	7	3.5	2	2.2	3	1.4	2	6.9		12	3.8	10	3.1	0.794	22	3.4

IAV: Influenza A virus; IBV: Influenza B virus; ICV: Influenza C virus; HRV: Human Rhinovirus; HADV:Human Adenovirus; HCoV-229E: Human Coronavirus 229E;HCoV-OC43: Human Coronavirus OC43; HPIV-1: Human Parainfluenza Virus-1; HPIV-2: Human Parainfluenza Virus-2; HPIV-3: Human Parainfluenza Virus-3; RSV-A: Respiratory Syncytial Virus-A; RSV-B: Respiratory Syncytial Virus-B;HMPV: Human Metapneumovirus; Boldface font indicates statistical.

# Case number

§ Percentage of detected virus in virus infected cases of each group

※ Detected virus

### Seasonal distribution of respiratory viruses

**Fig 1:**
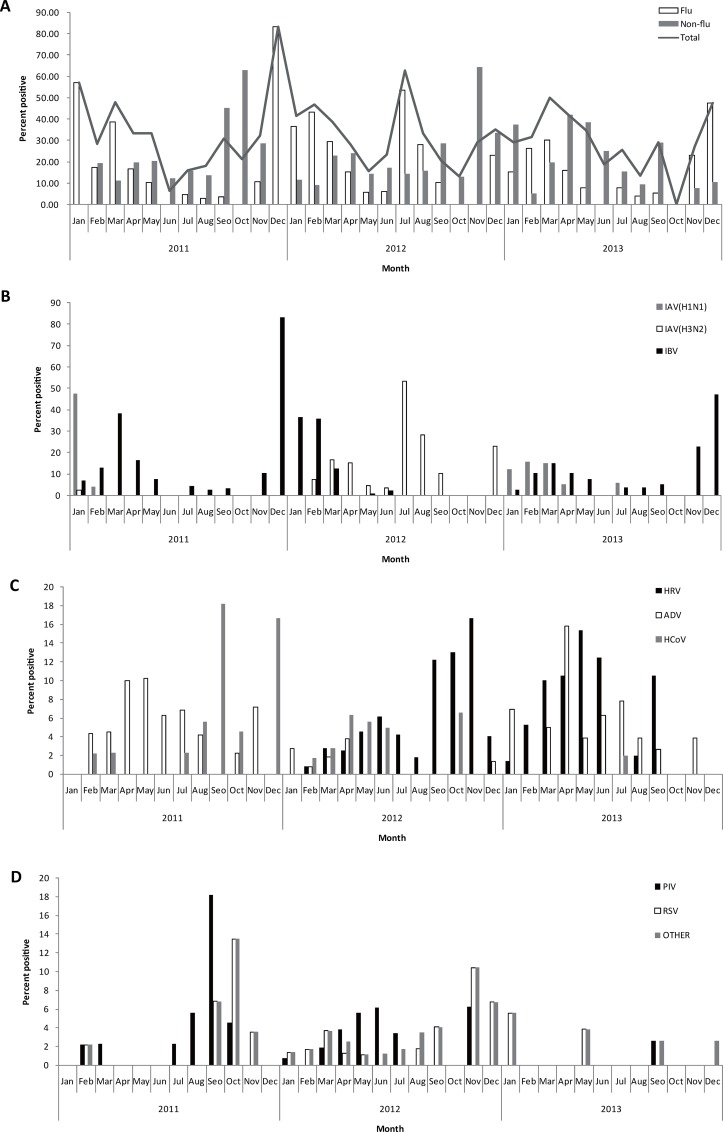
Monthly percentage of ILI outpatients positive for viral etiology in Shanghai, 2011–2013. A shows the monthly percentage of ILI outpatients positive for influenza virus, non-influenza virus and positive for any one of the studied respiratory viruses. B shows the monthly percentage of ILI outpatients positive for influenza subtypes. C shows the monthly percentage of ILI outpatients positive for HRV, HADV and HCoV. D shows the monthly percentage of ILI outpatients positive for HPIV, RSV and other respiratory viruses.

The characteristics of the seasonal patterns of respiratory viral pathogens were identified in our study. The monthly distribution of viruses is shown in Fig. 1A, 1B, 1C and 1D. We observed a winter peak in the detection rate in ILI patients during 3 years of surveillance and a summer peak in 2012. Influenza virus was the most commonly detected agent in the three winter seasons (i.e., 2010/2011, 2011/2012 and 2012/2013) and in one summer season (2012)(Fig. 1A and 1B). The results showed that IAV and IBV circulated from year to year in Shanghai (Fig. 1B).

HRV (not detected in 2011) was the second most frequently detected viral agent presenting during 2012 and 2013. It peaked in September-November in 2012 but showed no seasonality in 2013(Fig. 1C).HADV and HCoV were other major viral pathogens of ILI. HADV infection presented predominantly in 2011 and 2013, while HCoV presented predominantly in 2011 and 2012, and one peak was observed in September (Fig. 1C). HPIV, RSV, and HMPV appeared sporadically during the study period but presented an epidemic peak in September-October of 2011(Fig. 1D). The results suggested that multiple respiratory viruses may circulate concurrently in the population and cause a large proportion (approximately 40%) of ILI.

### Gender and age distribution of respiratory viral etiology

The distribution of respiratory viral pathogens from confirmed cases tested by RT-PCR by age and gender is shown in Table 3. Respiratory viruses were detected in 314(49.2%) of the male patients and 324(50.8%) of the female patients. There was no gender difference in susceptibility to ILI in Shanghai (Table 3).

IAV and IBV were most frequently identified in all age groups. The proportion of samples positive for IAV was higher in subjects 15–24 and 25–59 years of age (44.0%, and 41.8%, respectively) than in other age groups. This difference was statistically significant (*p* = 0.001).HCoV-229Eand HCoV-OC43 were detected frequently (13.3%, and 7.6%, respectively) in children less than 5 years of age. These values differed significantly from the corresponding values for other age groups (*p* = 0.002 and *p* = 0.01, respectively).HPMV and HBoV were only detected among subjects less than 15 years of age. HPIV was detected more frequently in subjects≥60 years of age compared with other age groups, but the difference between these frequencies was not statistically significant (Table 3).

### Factors Associated with Clinical Signs of ILI

The odds of having symptoms of cough, sore throat, running nose, fatigue, headache and temperature >39°C were higher in influenza virus-infected outpatients than in others, with odds ratios [ORs] of 1.99, 95% CI 1.60–2.47, *p<*0.001; 1.22, 95% CI 1.01–1.48,*p* = 0.038; 1.62, 95%CI 1.34–1.96, *p*<0.001; 1.25, 95%CI 1.02–1.53, *p* = 0.028; 1.24, 95%CI 1.01–1.52; and 1.40, 95%CI1.07–1.83, *p* = 0.015, respectively. Compared with the odds in patients ≥5 years of age, the odds of having a cough or running nose were higher for those in other age groups(OR3.22,95% CI 2.34–4.42; *p*<0.001;1.90,95% CI1.50–2.41,*p*<0.001,respectively). However, the odds of having a sore throat, fatigue or headache were higher for patients ≥5 years of age (OR2.56, 95%CI2.0–3.23, *p*<0.001; 50, 95%CI 20–100, *p*<0.001; and 100, 95%CI 20–333, *p*<0.001, respectively (Table 4).

**Table 4:** Results of multivariate analyses for factors associated with 6 clinical signs of virus positive in outpatients, Shanghai, China*, 2011–2013.

	Clinical sign of ILI§.								
Factors	Cough			Sore throat			Running nose		
	Yes, n = 494	No, n = 144	OR(95% CI),p value	Yes, n = 347	No, n = 291	OR(95% CI),p value	Yes, n = 309	No, n = 329	OR(95% CI),p value
other viral positive	174 (35.2)	71 (49.3)	Ref	133 (38.3)	112 (38.5)	Ref	109 (35.3)	136 (41.3)	Ref
Flu viral positive	320 (64.8)	73 (50.7)	**1.99(1.60–2.47),<0.001**	214 (61.7)	179 (61.5)	**1.22(1.01–1.48), 0.038**	200 (64.7)	193 (58.7)	**1.62(1.34–1.96), <0.001**
Age<5y	95 19.2)	10 (6.9)	**3.22(2.34–4.42), <0.001**	39 (11.2)	66 (22.7)	**0.39(0.31–0.50), <0.001**	62 (20.1)	43 (13.1)	**1.90(1.50–2.41), <0.001**
Male	243 (49.2)	71 (49.3)	1.08(0.89–1.31),0.432	169 (48.7)	145 (49.8)	0.96(0.81–1.15),0.682	152 (49.2)	162 (49.2)	0.99(0.83–1.19),0.907
Interval 2d#	38 7.7	10(6.9)	1.38(0.94–2.01),0.102	298.4	196.5	**1.71(1.21–2.42), 0.002**	247.8	247.3	0.99(0.70–1.40),0.955
Factors	Fatigue			Headache			Temperature※		
	Yes, n = 228	No, n = 410	OR(95% CI),p value	Yes, n = 199	No, n = 439	OR(95% CI),p value	>39°C, n = 102	≤39°C, n = 534	OR(95% CI),p value
other viral positive	67 (29.4)	178 (43.4)	Ref	60 (30.2)	185 (42.1)	Ref	2322.5	22241.6	Ref
Flu viral positive	161 (70.6)	232 (56.6)	**1.25(1.02–1.53), 0.028**	139 (69.8)	254 (57.9)	**1.24(1.01–1.52),0.043**	7977.5	31258.4	**1.40(1.07–1.83),0.015**
Age<5y	10.4	104(25.4)	**0.020.01–0.05), <0.001**	1 (0.5)	104(23.7)	**0.010.003–0.05), <0.001**	1918.6	8616.1	**1.31(0.95–1.82), 0.104**
Male	111 (48.7)	203(49.5)	0.96(0.79–1.16),0.662	99 (49.7)	215 (49.0)	0.92(0.76–1.12),0.413	45 (44.1)	268 (50.2)	0.95(0.73–1.23),0.682
Interval 2d#	33 (14.5)	15 (3.7)	**3.15(2.24–4.43),<0.001**	26 (13.1)	225.0	**2.04(1.45–2.87), <0.001**	87.8	407.5	0.830.49–1.390.474

*Six multivariate unconditional logistic regression analyses were separately conducted to examine factors for each clinical sign of ILI. ILI outpatients from whom influenza viral were detected positive by RT-PCR as confirmed influenza patients, and from whom other respiratory viral were detected positive by RT-PCR as confirmed other respiratory viral infection patients. Boldface font indicates statistical. OR, odds ratio; Ref, reference.

§Data are number (%) outpatients unless otherwise indicated.

#Interval between illness onset and hospital visit.

※Total of 2(0.2%)outpatients missing information on temperature results in the Influenza virus positive ILI-case group.

Multiplex RT-PCR is considered to be one of the most sensitive and specific methods for the diagnosis of respiratory virus infections [13, 14]. The Seeplex Respiratory Virus Detection assay (Seegene Inc., Seoul, Korea) was introduced to the respiratory research community in 2007 and can efficiently differentiate 12 respiratory viruses[10]. The application of multiplex design RT-PCR allows the simultaneous detection of multiple infections. In our study, multiplex viral agents, including IV (A and B), RSV (A and B), HPIV1-3, HRV, HCoVs (229E and OC43), HADV and HMPV were analyzed in a large population with ILI. To our knowledge, this is the first study of respiratory virus etiology with ILI in both children and adults in Shanghai. The overall detection rate of respiratory virus was 32.4%in the 3 years of laboratory-based surveillance. All of the 12 respiratory viruses were detected. Influenza virus was the most commonly detected agent in both children and adults; other viruses were present at a frequency less than 4.0%.

We observed a winter peak in the detection rate in ILI patients during 3 years of surveillance and a summer peak in 2012.Similar results have been reported in other studies in South China [15]. However, the ILI incidence only peaked during winter in Beijing and North China [6]. HCoV, HADV, and HRV were more frequently detected in children than in adults; HMPV and HBoV were only detected in children less than 15 years of age. Coughs, sore throats, running noses, fatigue, headache and temperatures >39°C were higher in influenza virus-infected outpatients than other respiratory virus-infected patients.

In our study, the investigation of the clinical characteristics of ILI showed that patients with IV experienced more coughs and temperatures >39°C than patients with other respiratory virus or those who were virus free. This result is consistent with the findings of previous research [6].

Children less than 5 years of age experienced more coughs, running noses and temperatures >39°C than adults. In the study by Friedman and Attia[16], the symptom triad of cough, headache, and pharyngitis was found to be a predictor of isolation of influenza virus in febrile children who were selected from emergency department patients at a tertiary pediatric center according to predetermined criteria indicating influenza infection. Respiratory viruses can cause influenza symptoms[17]. There is inconsistency about whether certain symptoms can be used to distinguish specific infections, and it is generally accepted that there are no symptoms specific to any viral infection. Some other studies have attempted to identify signs or symptoms specifically associated with influenza virus or other viruses [18, 19], but no definitive conclusions have been drawn.

During the study period, 32.4% of the specimens were positive for at least one virus. This result is consistent with the results of several previous studies that found percentages between 27–34.6% [6, 20–22], but it is lower than those reported by several investigators who found percentages between 48.7–59.5%[23, 24]. There are multiple explanations for these differences. The differences could be due to true differences in the overall burden, to differences among study populations, or to detection methods that differed from among studies. It is difficult to compare the results, as the reported data were obtained with different detection methods or PCR primers [21, 25]. Future comparative studies to evaluate the sensitivity and specificity of these detection methods should clarify this issue. Second, the infection rates may vary with geographical location and with the particular period chosen for testing [25, 26]. Third, the patient population and its environment may influence the results. In our study, up to 1332 specimens were negative in RT-PCR although all of them matched well with the inclusion criteria for ILI. Negative findings could have resulted from the low load of viral material in samples, or to infection with bacteria or other viruses such as enterovirus.

The seasonal pattern of ILI cases in Shanghai was quite unique according to our study.

Results differ markedly among studies in terms of the respiratory viruses most commonly found among studies. As in many previous studies [4, 6, 7, 20, 27–30], influenza A and B virus were the most commonly detected viral pathogens in outpatients with ILI in our region. An epidemic of influenza virus usually occurs in the winter. However, an epidemic peak of IAV infection was observed in the summer of 2012. More patients with influenza-like illness were observed during this period. This result was consistent with the Chinese National Influenza Center (CNIC) (2012 2013) China flu Weekly Report[31]. In addition, HRV, RSV, HADV, HCoV, HPIV and HMPV were identified from outpatient specimens and contributed collectively to 38.4% of all ILI laboratory-confirmed cases in our study. The detection rate of influenza virus was significantly higher among adult patients, whereas other viruses, such as HADV, HCoV, and HMPV, were prevalent among children less than 15 years of age. These findings are consistent with reports from other studies [5, 32–34].

HRV was not present in 2011, but it was the second most frequently detected viral agent in both children and adults in 2012 and 2013, representing approximately 10% of all positive cases. The positive rate (3.1%) was lower than that found by previous studies, which detected HRV in 10% to >40% of respiratory infections[29, 35]. HRV may circulate year-round in our region, a result found by previous studies [20, 25, 28]. RSV-positive samples occurred at a low level in our study. This finding contrasts with other studies [27, 36], where the predominant virus detected in children less than 5 years of age was RSV. The reason for this difference is that we did not include hospitalized children, as RSV has been shown to be a common cause of lower respiratory tract infection in children admitted to the hospital[5, 37, 38]. Previous studies on RSV infections have primarily been conducted in hospitalized elderly adults with medical conditions such as cardiopulmonary diseases[39] that may predispose them to viral infections that are relatively uncommon in the general population.

In our study, a total of 22 samples (3.5% of the total number of RT-PCR positive cases) revealed the presence of co-infections. In co-infection cases, influenza A virus, PIV, and RSV were found together most frequently, at rates of 36.4% (8/22). Previous studies have reported that co-infections were associated with more severe signs than mono-infections [40, 41]. Children were found to be more likely to be co-infected than adults in the present study. However, the subsequent clinical conditions of ILI illness patients were not obtained, and thus the association between co-infections and severe signs cannot be analyzed. We will address such research themes in the future.

This study has limitations. First, no testing for other etiologies (such as enterovirus or bacteria) of acute respiratory illness was performed. As is generally known, respiratory viruses, bacteria and other microorganisms can cause respiratory illness with influenza-like symptoms. Without doubt, other microorganisms could have been additional pathogens in the negative specimens, and our results may underestimate the role of virus infection. Second, the histories of influenza vaccine in ILI outpatients were not obtained. Thus, the analysis of clinical characteristics in ILI patients may be biased. Third, we did not collect all ILI cases presenting at the above two sentinel sites from Monday through Sunday. Facility staffs were involved in the project on a voluntary basis, with frequent shifts of personnel to other facilities.

In conclusion, the spectrum, seasonality, age distribution and clinical associations of respiratory virus infections in children and adults with influenza-like illness were analyzed in Shanghai for the first time in this study. To a certain extent, the findings can provide baseline data for evaluating the burden of respiratory virus infection in children and adults in Shanghai. It also can provide clinicians with helpful information about the etiological patterns of outpatients presenting with complaints of acute respiratory symptoms, but further studies should be conducted, and longer-term laboratory-based surveillance would give a better picture of the etiological characteristics of ILI.

## Supporting Information

S1 Dataset Detailed information for all the case involved in our study. (XLS)

S1 File Editorial certificate of this manuscript. (PDF)

## References

[1] Heijnen ML, Dorigo-Zetsma JW, Bartelds AI, Wilbrink B, Sprenger MJ. Surveillance of respiratory pathogens and influenza-like illnesses in general practices—The Netherlands, winter 1997–98. Euro Surveill 1999;4: 81–84. 12631895 10.2807/esm.04.07.00054-en

[2] Laguna-Torres VA, Sanchez-Largaespada JF, Lorenzana I, Forshey B, Aguilar P, Jimenez M, et al Influenza and other respiratory viruses in three Central American countries. Influenza Other Respir Viruses 2011;5: 123–134. 10.1111/j.1750-2659.2010.00182.x 21306576 PMC4942008

[3] Mackay IM. Human rhinoviruses: the cold wars resume. J Clin Virol 2008;42: 297–320. 10.1016/j.jcv.2008.04.002 18502684 PMC7108405

[4] Tokarz R, Kapoor V, Wu W, Lurio J, Jain K, Mostashari F, et al Longitudinal molecular microbial analysis of influenza-like illness in New York City, May 2009 through May 2010. Virol J 2011;8: 288 10.1186/1743-422X-8-288 21658237 PMC3121709

[5] Lina B, Valette M, Foray S, Luciani J, Stagnara J, See DM, et al Surveillance of community-acquired viral infections due to respiratory viruses in Rhone-Alpes (France) during winter 1994 to 1995. J Clin Microbiol 1996;34: 3007–3011. 8940439 10.1128/jcm.34.12.3007-3011.1996 PMC229450

[6] Yang X, Yao Y, Chen M, Yang X, Xie Y, Liu Y, et al Etiology and clinical characteristics of influenza-like illness (ILI) in outpatients in Beijing, June 2010 to May 2011. PLoS One 2012;7: e28786 10.1371/journal.pone.0028786 22238581 PMC3251557

[7] Puzelli S, Valdarchi C, Ciotti M, Dorrucci M, Farchi F, Babakir-Mina M, et al Viral causes of influenza-like illness: Insight from a study during the winters 2004–2007. J Med Virol 2009;81: 2066–2071. 10.1002/jmv.21610 19856468

[8] Mahony J, Chong S, Merante F, Yaghoubian S, Sinha T, Lisle C, et al Development of a respiratory virus panel test for detection of twenty human respiratory viruses by use of multiplex PCR and a fluid microbead-based assay. J Clin Microbiol 2007;45: 2965–2970. 17596360 10.1128/JCM.02436-06 PMC2045291

[9] Templeton KE, Scheltinga SA, Beersma MF, Kroes AC, Claas EC. Rapid and sensitive method using multiplex real-time PCR for diagnosis of infections by influenza a and influenza B viruses, respiratory syncytial virus, and parainfluenza viruses 1, 2, 3, and 4. J Clin Microbiol 2004;42: 1564–1569. 15071005 10.1128/JCM.42.4.1564-1569.2004 PMC387552

[10] Kim SR, Ki CS, Lee NY. Rapid detection and identification of 12 respiratory viruses using a dual priming oligonucleotide system-based multiplex PCR assay. J Virol Methods 2009;156: 111–116. 10.1016/j.jviromet.2008.11.007 19063921 PMC7112863

[11] Yang P, Duan W, Lv M, Shi W, Peng X, Wang X, et al Review of an influenza surveillance system, Beijing, People's Republic of China. Emerg Infect Dis 2009;15: 1603–1608. 10.3201/eid1510.081040 19861053 PMC2866378

[12] Centers for Disease Control and Prevention Overview of influenza surveillance in the United States, 2007 vol. 2014, 2014.

[13] Deng J, Ma Z, Huang W, Li C, Wang H, Zheng Y, et al Respiratory virus multiplex RT-PCR assay sensitivities and influence factors in hospitalized children with lower respiratory tract infections. Virol Sin 2013;28: 97–102. 10.1007/s12250-013-3312-y 23575731 PMC7090616

[14] Lassauniere R, Kresfelder T, Venter M. A novel multiplex real-time RT-PCR assay with FRET hybridization probes for the detection and quantitation of 13 respiratory viruses. J Virol Methods 2010;165: 254–260. 10.1016/j.jviromet.2010.02.005 20153377 PMC7112774

[15] Huo X, Qin Y, Qi X, Zu R, Tang F, Li L, et al Surveillance of 16 respiratory viruses in patients with influenza-like illness in Nanjing, China. J Med Virol 2012;84: 1980–1984. 10.1002/jmv.23401 23080506 PMC7166984

[16] Friedman MJ, Attia MW. Clinical predictors of influenza in children. Arch Pediatr Adolesc Med 2004;158: 391–394. 15066881 10.1001/archpedi.158.4.391

[17] Hasman H, Pachucki CT, Unal A, Nguyen D, Devlin T, Peeples ME, et al Aetiology of influenza-like illness in adults includes parainfluenzavirus type 4. J Med Microbiol 2009;58: 408–413. 10.1099/jmm.0.006098-0 19273634 PMC2778239

[18] Boivin G, Hardy I, Tellier G, Maziade J. Predicting influenza infections during epidemics with use of a clinical case definition. Clin Infect Dis 2000;31: 1166–1169. 11073747 10.1086/317425

[19] Peltola V, Reunanen T, Ziegler T, Silvennoinen H, Heikkinen T. Accuracy of clinical diagnosis of influenza in outpatient children. Clin Infect Dis 2005;41: 1198–1200. 16163640 10.1086/444508

[20] Ren L, Gonzalez R, Wang Z, Xiang Z, Wang Y, Zhou H, et al Prevalence of human respiratory viruses in adults with acute respiratory tract infections in Beijing, 2005–2007. Clin Microbiol Infect 2009;15: 1146–1153. 10.1111/j.1469-0691.2009.02746.x 19456830 PMC7129754

[21] Druce J, Tran T, Kelly H, Kaye M, Chibo D, Kostecki R, et al Laboratory diagnosis and surveillance of human respiratory viruses by PCR in Victoria, Australia, 2002–2003. J Med Virol 2005;75: 122–129. 15543580 10.1002/jmv.20246 PMC7166941

[22] Pierangeli A, Gentile M, Di Marco P, Pagnotti P, Scagnolari C, Trombetti S, et al Detection and typing by molecular techniques of respiratory viruses in children hospitalized for acute respiratory infection in Rome, Italy. J Med Virol 2007;79: 463–468. 17311326 10.1002/jmv.20832 PMC7166338

[23] Ju X, Fang Q, Zhang J, Xu A, Liang L, Ke C. Viral etiology of influenza-like illnesses in Huizhou, China, from 2011 to 2013. Arch Virol 2014. 10.1007/s00705-014-2035-1 PMC7086676 24610554

[24] Wang W, Cavailler P, Ren P, Zhang J, Dong W, Yan H, et al Molecular monitoring of causative viruses in child acute respiratory infection in endemo-epidemic situations in Shanghai. J Clin Virol 2010;49: 211–218. 10.1016/j.jcv.2010.08.005 20855230 PMC7185670

[25] Bellei N, Carraro E, Perosa A, Watanabe A, Arruda E, Granato C. Acute respiratory infection and influenza-like illness viral etiologies in Brazilian adults. J Med Virol 2008;80: 1824–1827. 10.1002/jmv.21295 18712837 PMC7166366

[26] Larcher C, Jeller V, Fischer H, Huemer HP. Prevalence of respiratory viruses, including newly identified viruses, in hospitalised children in Austria. Eur J Clin Microbiol Infect Dis 2006;25: 681–686. 17036151 10.1007/s10096-006-0214-z PMC7087607

[27] Niang MN, Diop OM, Sarr FD, Goudiaby D, Malou-Sompy H, Ndiaye K, et al Viral etiology of respiratory infections in children under 5 years old living in tropical rural areas of Senegal: The EVIRA project. J Med Virol 2010;82: 866–872. 10.1002/jmv.21665 20336732 PMC7166331

[28] Razanajatovo NH, Richard V, Hoffmann J, Reynes JM, Razafitrimo GM, Randremanana RV, et al Viral etiology of influenza-like illnesses in Antananarivo, Madagascar, July 2008 to June 2009. PLoS One 2011;6: e17579 10.1371/journal.pone.0017579 21390235 PMC3048401

[29] Njouom R, Yekwa EL, Cappy P, Vabret A, Boisier P, Rousset D. Viral etiology of influenza-like illnesses in Cameroon, January-December 2009. J Infect Dis 2012;206 Suppl 1: S29–35. 10.1093/infdis/jis573 23169968 PMC7107314

[30] Li H, Wei Q, Tan A, Wang L. Epidemiological analysis of respiratory viral etiology for influenza-like illness during 2010 in Zhuhai, China. Virol J 2013;10: 143 10.1186/1743-422X-10-143 23651577 PMC3655035

[31] Chinese National Influenza Center (CNIC) (2011 2013) China flu Weekly Report, vol. 2014, 2014.

[32] Michelow IC, Olsen K, Lozano J, Rollins NK, Duffy LB, Ziegler T, et al Epidemiology and clinical characteristics of community-acquired pneumonia in hospitalized children. Pediatrics 2004;113: 701–707. 15060215 10.1542/peds.113.4.701

[33] Nichols WG, Peck Campbell AJ, Boeckh M. Respiratory viruses other than influenza virus: impact and therapeutic advances. Clin Microbiol Rev 2008;21: 274–290, table of contents. 10.1128/CMR.00045-07 18400797 PMC2292575

[34] O'Riordan S, Barton M, Yau Y, Read SE, Allen U, Tran D. Risk factors and outcomes among children admitted to hospital with pandemic H1N1 influenza. CMAJ 2010;182: 39–44. 10.1503/cmaj.091724 19926677 PMC2802603

[35] Waner JL. Mixed viral infections: detection and management. Clin Microbiol Rev 1994;7: 143–151. 8055464 10.1128/cmr.7.2.143 PMC358314

[36] Zhang G, Hu Y, Wang H, Zhang L, Bao Y, Zhou X. High incidence of multiple viral infections identified in upper respiratory tract infected children under three years of age in Shanghai, China. PLoS One 2012;7: e44568 10.1371/journal.pone.0044568 22970251 PMC3436764

[37] Ranmuthugala G, Brown L, Lidbury BA. Respiratory syncytial virus—the unrecognised cause of health and economic burden among young children in Australia. Commun Dis Intell Q Rep 2011;35: 177–184. 22010512 10.33321/cdi.2011.35.15

[38] Stockman LJ, Curns AT, Anderson LJ, Fischer-Langley G. Respiratory syncytial virus-associated hospitalizations among infants and young children in the United States, 1997–2006. Pediatr Infect Dis J 2012;31: 5–9. 10.1097/INF.0b013e31822e68e6 21817948

[39] Murata Y. Respiratory syncytial virus infection in adults. Curr Opin Pulm Med 2008;14: 235–240. 10.1097/MCP.0b013e3282f79651 18427247

[40] Wolf DG, Greenberg D, Kalkstein D, Shemer-Avni Y, Givon-Lavi N, Saleh N, et al Comparison of human metapneumovirus, respiratory syncytial virus and influenza A virus lower respiratory tract infections in hospitalized young children. Pediatr Infect Dis J 2006;25: 320–324. 16567983 10.1097/01.inf.0000207395.80657.cf

[41] Frobert E, Escuret V, Javouhey E, Casalegno JS, Bouscambert-Duchamp M, Moulinier C, et al Respiratory viruses in children admitted to hospital intensive care units: evaluating the CLART(R) Pneumovir DNA array. J Med Virol 2011;83: 150–155. 10.1002/jmv.21932 21108353 PMC7167182

