# Supplementary material for: The Clinical and Etiological Characteristics of Influenza-Like Illness (ILI) in Outpatients in Shanghai, China, 2011 to 2013
Source: PLoS One. 2015 Mar 30;10(3):e0119513. doi: 10.1371/journal.pone.0119513 (PMC4379014; doi:10.1371/journal.pone.0119513)
Supplement: S1 File — (PDF) [file pone.0119513.s002.pdf]

AMERICAN JOURNAL EXPERTS

EDITORIAL CERTIFICATE

This document certifies that the manuscript listed below was edited for proper English language, grammar, punctuation, spelling, and overall style by one or more of the highly qualified native English speaking editors at American Journal Experts.

Manuscript title:

Clinical and etiological characteristics of influenza-like illness (ILI) in outpatients in Shanghai, China, 2011 to 2013

Authors:

Yifei Fu, Lifeng Pan, Qiao Sun, Linying Zhu, Qing Liu, Ping Ma, Huifang Qiu, Weiping Zhu, Chuchu Ye, Caoyi Xue, Yuanping Wang

Date Issued:

January 23, 2015

Certificate Verification Key:

BCB9-4447-E689-12FE-28EC

This certificate may be verified at www.aje.com/certificate. This document certifies that the manuscript listed above was edited for proper English language, grammar, punctuation, spelling, and overall style by one or more of the highly qualified native English speaking editors at American Journal Experts. Neither the research content nor the authors' intentions were altered in any way during the editing process. Documents receiving this certification should be English-ready for publication; however, the author has the ability to accept or reject our suggestions and changes. To verify the final AJE edited version, please visit our verification page. If you have any questions or concerns about this edited document, please contact American Journal Experts at support@aje.com.

American Journal Experts provides a range of editing, translation and manuscript services for researchers and publishers around the world. Our top-quality PhD editors are all native English speakers from America's top universities. Our editors come from nearly every research field and possess the highest qualifications to edit research manuscripts written by non-native English speakers. For more information about our company, services and partner discounts, please visit www.aje.com.
